# Psychotherapy Augmentation through Preconscious Priming

**DOI:** 10.3389/fpsyt.2013.00015

**Published:** 2013-03-18

**Authors:** François Borgeat, Kieron O’Connor, Danielle Amado, Marie-Ève St-Pierre-Delorme

**Affiliations:** ^1^Fernand-Seguin Research Centre, Institut universitaire en santé mentale de MontréalMontreal, QC, Canada; ^2^Department of Psychiatry, University of MontrealMontréal, QC, Canada; ^3^University of LausanneLausanne, Switzerland

**Keywords:** psychotherapy augmentation, priming, preconscious, cognitions, social phobia

## Abstract

**Objective:** To test the hypothesis that repeated preconscious (masked) priming of personalized positive cognitions could augment cognitive change and facilitate achievement of patients’ goals following a therapy.

**Methods:** Twenty social phobic patients (13 women) completed a 36-weeks study beginning by 12 weeks of group behavioral therapy. After the therapy, they received 6 weeks of preconscious priming and 6 weeks of a control procedure in a randomized cross-over design. The Priming condition involved listening twice daily with a passive attitude to a recording of individualized formulations of appropriate cognitions and attitudes masked by music. The Control condition involved listening to an indistinguishable recording where the formulations had been replaced by random numbers. Changes in social cognitions were measured by the Social Interaction Self Statements Test (SISST).

**Results:** Patients improved following therapy. The Priming procedure was associated with increased positive cognitions and decreased negative cognitions on the SISST while the Control procedure was not. The Priming procedure induced more cognitive change when applied immediately after the group therapy.

**Conclusion:** An effect of priming was observed on social phobia related cognitions in the expected direction. This self administered addition to a therapy could be seen as an augmentation strategy.

## Introduction

The purpose of this study is to evaluate the potential augmenting or facilitating effects of a perceptual strategy on a psychotherapeutic process. The study used the context of a group behavioral treatment of social phobia. The strategy is based on preconscious perception, masked priming research, and cognitive analysis as practised in cognitive-behavioral therapy (CBT). This paper will focus on the description and testing of this novel (to our knowledge) strategy.

### Does psychotherapy need augmentation of its effectiveness?

Obviously not always, but not infrequently either. The effectiveness of psychotherapy has been well established (Smith and Glass, 1977) even if polemics continue over the comparative merits of different approaches (INSERM, 2004). In social phobia, Davidson et al. (2004) have reported significant improvement with CBT or fluoxetine or both but also many residual symptoms after 14 weeks of treatment.

In clinical practice, limited or slow change in psychotherapy can occur even in the presence of a positive patient-therapist alliance. One of the factors could be that psychotherapists face the very difficult task of helping patients to solve mainly emotional and irrational problems and trying to change more or less conscious habits. Treatment methods are almost always based on rational and conscious communication. CBT further implies that cognitions underlie symptoms and that they are accessible to awareness and modifiable through reasoning. Psychotherapies have usually emphasized the content of therapeutic communication much more than the method or the channel through which this information could be communicated to the patient to maximize effectiveness. Indeed, it is important to highlight internal psychic mechanisms that are thought to underlie the symptoms reported and to bring awareness to patients. The strategies to communicate awareness effectively to patients and to help them change their psychic mechanisms have remained contested.

An additional observation pertinent both to psychotherapy and to this study is the tendency of psychotherapists to identify strongly with their preferred theories and, as a result, attempt to solve clinical difficulties mainly through knowledge or techniques related to those theories. If this tendency can improve the coherence of their approach, it can also result in doing more of the same thing, sometimes in spite of limited progress. This also impedes a potentially useful integration of different strategies and the exploration of fields of knowledge exterior to psychotherapy, which could perhaps impact therapeutic methods and possibly help or accelerate change in the course of psychotherapy. The strategy reported here borrows from research on preconscious perception and on masked priming, both parts of experimental paradigms developed outside the domain of psychotherapy.

### What is augmentation?

Augmentation strategies have become common in psychopharmacology but have very seldom been proposed in psychotherapy. The only exception to our knowledge is the use of d-cycloserine, a putative cognitive enhancer, to increase the effect of exposure therapy in the treatment of phobic individuals (Ressler et al., 2004). The term augmentation has also been used to describe combined treatment involving the association of pharmacotherapy and psychotherapy. In fact, those types of combined treatments, while useful and often recommended in practice guidelines, are usually simple juxtapositions of two effective treatments hopefully having additive effects. In our view, such juxtapositions or addition of treatments are not really psychotherapy augmentation strategies since they do not attempt to modify or facilitate the psychotherapeutic process. The term should probably be reserved to strategies aiming to augment or accelerate the psychological change induced by psychotherapy. The augmentation strategy could be biological or, as described in this study, psychological.

The questioned strategy assessed in this study is: can conscious accessibility to more appropriate cognitions and imagery be increased by a repeated priming procedure? Recent research has indicated that anxious and depressed patients show restricted vividness when attempting to generate positive prospective scenarios as compared with controls (Morina et al., 2011), and that anxious patients reported greater ability to generate negative than positive scenarios. Hence increasing availability of more positive imagery and cognitions could, if feasible, constitute a useful focus for a strategy aiming to augment the effects of a CBT approach of anxiety or depression. A technique called imagery rehearsal therapy has been shown a useful short-term addition to the treatment of PTSD with a potential to reduce nightmares by modifying their imagery, a goal similar to the aim of this study (Krakow et al., 2001; Nappi et al., 2010).

### What is preconscious priming?

Two concepts are involved: preconscious and priming. Over the last decades, our group has been concerned with preconscious (or subliminal) perception, initially by measuring the psychophysiological effects of preconscious stimuli (Borgeat and Goulet, 1983; Borgeat et al., 1985, 1989). Recently the neurophysiological activity of various levels of non-conscious processing has been clarified suggesting a taxonomy that distinguishes between subliminal, preconscious, and conscious processing (Dehaene et al., 2006). More generally the scholarly review of preconscious processing by Dixon (1981) remains useful and gives an idea of the amount of data and evidence on this phenomenon even though it was written 30 years ago.

The augmentation strategy proposed in this paper is related to research findings in various areas like semantic priming and masked priming (Liddell et al., 2004; Kouider and Dupoux, 2005; Ruiz-Padial et al., 2005), unattended information (Corteen and Wood, 1972), preconscious processing (Elliott and Dolan, 1998), and subliminal psychodynamic activation (Silverman et al., 1982). That last domain of research has yielded some of the rare attempts to apply subliminal perception in clinical treatment, for example to improve eating disorders and by showing the possible therapeutic effects of stimulating oneness fantasy (Silverman et al., 1982; Waller and Barter, 2005). Swingle (1992) has proposed several case studies and the first guide of possible subliminal procedures in clinical settings.

Clear evidence exists that anxiety can be triggered by stimuli remaining outside of awareness. Ohman and Soares (1994) have elicited phobic responses to masked fearful pictures even if they were not recognized consciously. The subliminal stimuli induced emotional and physiological responses. Studies on perceptual defense also showed that preconscious stimuli could slow or accelerate the perceptual process according to their emotional valence before they achieve awareness (Borgeat et al., 1997; Poloni et al., 2003). Preconscious presentation of stimuli has also been used to study emotional processing biases occurring automatically below the level of conscious awareness. In major depressive disorder, a negative bias toward sad faces has been demonstrated with larger amygdala responses on fMRI (Victor et al., 2010).

Priming is a phenomenon and a research method in cognitive psychology showing that perception of targets stimuli is influenced or “primed” by previous stimuli. For instance, if a subject has been exposed to bird related words or images playing here the role of primes, he will be faster to identify bird stimuli than if he had been exposed to car related words or pictures. This indicates that previous information is used to facilitate the processing of new information, probably by making the semantic network of bird related memories more readily available, as in our example. This phenomenon, which makes sense for effective and quick information processing, has been termed “entry opening” (Forster et al., 2003). It has been applied mainly to the study of mechanisms involved in word recognition. Obviously there is no reason why that phenomenon would be restricted to laboratory experiments on word recognition and it probably reveals more general mechanisms involved in accessing memories. Priming has also been observed with masked primes that were not perceived consciously. A masked prime is a briefly presented stimulus immediately covered by another stimulus acting as a mask (e.g., a grid) and preventing the conscious perception of the prime. Interestingly, masked primes appear more effective that unmasked ones to produce affective effects and subliminal affective priming appears to produce long-lasting effects of at least 24 h (Masson and Bodner, 2003; Sweeny et al., 2009).

Consciously, the patient is attempting to modify his own repertoire of irrational thoughts and behaviors, and related affective responses, but usually through rational discussion. The patient would like to access and use more easily, in anxiety provoking situations, a more comfortable repertoire of cognitions and emotional responses. In this study, repeated preconscious priming attempts to facilitate access to such a repertoire.

Most priming procedures, but not all of them, use the visual perceptual modality. In contrast, most therapeutic procedures are based on the auditory modality, as are most relaxation and meditation techniques that use verbal instructions. Over several years, our group has developed and studied methods of subliminal or masked auditory stimulation that can be considered as procedures of masked auditory priming. Typically, auditory stimuli presented at 20 dbs are mixed with a masking sound, e.g., a white noise of 40 dbs in a laboratory or a relaxing music in a clinical situation. With such a mix, the stimuli are not identified by subjects but have been shown to produce measurable effects (Borgeat et al., 1985). Modification of physiological responses to stressors has been observed, especially enhanced responses (Borgeat et al., 1989). Influence on the subjective activation level of a person has also been demonstrated. Masked auditory stimuli have been shown to induce more mental images than other techniques when used for relaxation (Borgeat, 1982). Subliminal sexual auditory or visual stimulation have been reported to increase erotic imagery, desire, and genital response (Borgeat et al., 1988; Ponseti and Bosinski, 2010). More generally those various studies have also indicated that the simple masking procedure developed in auditory subliminal or preconscious stimulation could produce reliable effects congruent with the content of the stimuli and that such a procedure could be easily translated to clinical situations.

### Why social phobia?

To test our strategy, the selection of a homogeneous group of patients appeared essential. Social phobia is a frequent and often disabling anxiety disorder. While the manifestations and symptoms of social phobia vary among individuals, there is always at its core an inordinate fear of judgment and humiliation. Negative self-imagery generating negative self-thoughts appears to constitute a key factor in this condition (Hirsch et al., 2006). The treatment of choice is behavioral, and all patients underwent a group behavioral treatment aimed to improve social functioning (Stravynski, 2007). In social phobia, what helps beyond exposure remains matter of debate and research. Among the usual components of CBT, more exposure appears related to more rapid positive cognitive changes while cognitive work was shown to contribute to more persistent decrease of social avoidance (Borgeat et al., 2009). Some unpublished pilot case studies of our group had indicated that social phobic patients making limited progress in CBT could respond to augmentation by preconscious priming.

This randomized controlled study assesses the effects of adding preconscious priming after a group behavioral therapy in an attempt to augment its effect or to facilitate therapeutic change in the direction of patient’s well-defined goals that remained partially unachieved. The underlying rationale is that the procedure could lead to priming of positive semantic and affective memories and as a result make new cognitive and affective repertoires more available to the patient, thus facilitating behavioral and emotional change in synergy with the psychotherapeutic work. Hence the hypothesis is that a personalized and repeated preconscious priming procedure would be associated to (1) a greater reduction of negative social cognitions and (2) to a greater increase of positive social cognitions than a control condition. A secondary research question attempts to evaluate the changes in social anxiety scores associated with the cognitive responses to the procedure.

## Materials and Methods

### Subjects

Thirty social phobic patients were evaluated and gave their informed consent to participate in the study which had been approved by the ethics committee of the Fernand-Seguin Research Centre of Montreal. Those informed consents are archived by the authors. Twenty-five patients were included on the basis of a DSM-IV main diagnosis of social phobia and a score of at least 50 on the Liebowitz Social Anxiety Scale (LSAS). Patients with another anxiety disorder as a primary condition or currently meeting DSM-IV criteria for any mood disorder were excluded. Patients with comorbid dysthymia were allowed to enter the study since their pre-therapy functional analysis revealed that dysthymia when present appeared more as a consequence of patients’ social anxiety and unsatisfactory interpersonal functioning. Patients with a lifetime history of any psychotic disorder or a recent (6 months) history of alcohol or substance abuse/dependence were also excluded.

### Measures

#### Primary outcome measure

The primary efficacy measure focused on social cognitions using the Social Interaction Self Statement Test (SISST) to measure the changes from the baseline (week 0) throughout the treatment and the follow-up. The SISST is a commonly used self-report test for social anxiety including 15 negative and 15 positive statements which the participants score from 1, “hardly ever had the thought” to 5, “very often had the thought” (Glass et al., 1982). The French validated version TAPIS was used (Yao et al., 1998).

#### Secondary outcome measures

The secondary outcome measures include the changes on the LSAS [Liebowitz, 1987 and the Fear of Negative Evaluation Scale (FNE) (Watson and Friend, 1969)].

The LSAS (Liebowitz, 1987) is a standard measure of social anxiety including 24 items rated on the level of anxiety and on the level of avoidance. Of the 24 items, 13 are concerned with performance anxiety and 11 with social situations.

Fear of Negative Evaluation Scale (Watson and Friend, 1969) is a 30-item true-false inventory concerning negative evaluations of self and social life. Test-retest reliability is 0.75 and internal consistency = 0.9.

### Procedure

The patients were firstly treated by means of a 12-week group behavior therapy aiming at improving social functioning (Stravynski et al., 2000). Groups included five to six patients. Typically, the session was divided in two: after a return of what they did during the week, patients learned through role-playing how to cope with their feared social situations. They received feed-back form their peers and the therapists. They were asked to repeat the worked social interaction in their real-life setting for the following week. Emphasis was put on improving social functioning (by achieving some specific targeted goals), rather than only reducing social anxiety *per se*. Therapy was conducted at the Fernand-Seguin Research Center of Montreal by a psychologist with 10 year experience in this approach (DA) assisted by a psychologist PhD candidate acting as co-therapist.

After the group therapy, they underwent the experimental procedure alternating preconscious priming and placebo during 6 weeks each for an additional 12 weeks. Finally they were followed for 12 weeks more afterward, for a total 36 weeks study.

The patients had been randomized at baseline to two groups corresponding to two sequences (Priming first followed by Control or the reverse sequence) and enrolled in a cross-over design whereby all patients received alternatively the two treatments (Priming and Control). The SISST was administered at six time points and these were the study variables.

The Priming condition involved listening to a recorded CD for 20 min twice a day in a relaxed setting with a passive attitude or before exposure to situations specifically associated to social anxiety of the subjects. The CD contained formulations of appropriate cognitions and attitudes masked by a calm music (JS Bach’s Goldberg Variations interpreted by Glenn Gould, BWV 988). The formulations are individualized as described in the procedure below. The Control condition involved listening to a recorded CD similar to the first one but in which the formulations had been replaced by random numbers.

The two tapes were indistinguishable: after a similar brief introduction suggesting relaxation and calmness, the personalized formulations or the random numbers were masked by the music mixed at an intensity of 20–30 dbs higher for the music. Subjects were told that one CD contained more of the personalized formulations elaborated with each of them as described below, while the other CD contained more general stress reducing suggestions and less of those personalized formulations.

Measures were taken at baseline (before group therapy), 6 weeks (mid-therapy), 12 weeks (end of group therapy and beginning of first CD for Priming or Control), 18 weeks (end of first CD and beginning of second CD), 24 weeks, and 36 weeks. The rater was blind to CDs’ content.

### Preconscious priming procedure

In spite of similar diagnoses, the focus of behavioral or cognitive-behavioral psychotherapy is always highly individualized and thus differs from patient to patient. Similarly the content of the masked stimuli presented in the priming procedure was also individualized for the 20 patients while obviously always focusing on cognitive aspects of social anxiety.

The procedure identical for all subjects followed six steps. Firstly, during a session of the second half of their 12 week group therapy focusing on interpersonal behavior (Stravynski, 2007), they met the principal investigator who explained the rationale and the various steps of the procedure and answered their questions. They were told that the procedure aimed at helping them to change their automatic anxious inner talk in the direction they wished and to render this alternative and more adaptive new inner talk more available and more automatic when needed. To prepare themselves they were asked to write down on a single sheet of paper the anxious inner talk they most wished to change. They were instructed to put down not only cognitions, but also memories, emotions, physiological reactions, and behavior.

Secondly, at the end of group therapy, each patient met individually with the principal investigator and the group therapist to select a remaining difficulty and define a specific goal of change. This goal was more explicitly defined by constructing what we came to call a semantic and affective network describing the connections between the actual dysfunctional cognitions, memories, and emotions with their behavioral or physiological consequences.

Thirdly, starting from that network of dysfunctional cognitions and emotions, an alternative network of more appropriate and desired cognitions, attitudes, and behavior was established to reflect patient’s goal of change, also in a close collaboration between patient, therapist, and experimenter. The task was to describe those two networks (the dysfunctional one and the desired one) on a single sheet of paper to ensure a clear and simple focus of desired change.

Fourthly, when this focus of change had become clear, simple formulations of these desired cognitions or attitudes were discussed until agreed upon. A first draft of these formulations was initiated during the meeting between patient, therapist, and experimenter and completed by an exchange of e-mails until the patient felt that these brief sentences reflected at best the kind of inner talk he wished to be more available especially in stressful situations. In addition, therapist and experimenter had to agree that the formulations were appropriate and directly related to the patient’s problem and goal of change. The task was to agree on a list of 10–15 brief sentences describing cognitions, states of mind, or attitudes that the patient wished to make his or her own. A few intuitive principles were applied. For instance, sentences or suggestions were to be written using simple and concrete words rather than abstract ones. They were to be written using positive words each of them reflecting as much as possible an aspect of the desired change. Negative formulations were avoided.

Fifthly, these formulations were recorded and mixed with a masking relaxing music (JS Bach’s Goldberg Variations interpreted by Glenn Gould, BWV 988) in a professional recording studio (MixArt, Montreal, QC, Canada). The sentences were read by the same experimenter for all subjects to ensure uniformity (as were the general relaxation suggestions at the beginning of all CDs and the random numbers of the placebo CDs). The intensity was 20–30 dbs higher for the music to ensure masking taking into account the natural variations in the music intensity. With this combination, the suggestions were masked at least to the level of a non-identifiable mumble. The mixes lasted approximately 20 min and were recorded on a CD. All CDs began by the same audible general suggestions for a pleasant experience, calmness, and relaxation for about 30 s. The CDs were given to patients for home use.

Sixthly, the patient was instructed to listen to this CD at least twice a day as a relaxation exercise and especially at bedtime when falling asleep and before or during challenging social situations. The patients were asked to adopt a passive attitude while listening to the recording and more specifically not to try to attend the masked messages but rather to focus effortlessly on the music.

### Statistical analysis

Analysis was undertaken using SPSS version 19. Effect sizes were calculated using η^2^. Measures taken from week 0 (beginning of group therapy) to 36 (end of follow-up) were considered in analysis. The effects of the two groups (group A and group B) corresponding respectively to the two sequences (Priming first followed by Control or the reverse sequence; abbreviated respectively to: priming first and Control first) were analyzed through two-tailed ANOVAs for repeated measures. The effects of the two interventions (Priming and Control) were also analyzed through paired *t*-tests.

## Results

### Baseline analysis

Twenty-five participants were included in the study. Two patients dropped-out during the group therapy for personal reasons (relocation). Twenty-three participants were included in the analyses: 10 men and 13 women. Mean age for the total sample was 38 years (SD = 7.95) and mean age for sequence Priming first followed by Control was 37 years (SD = 8.55) and sequence Control first followed by Priming was 39 years (SD = 7.45). There was no statistical age difference between the two sequences: *F*(1,21) = 0.62, *p* = 0.44.

### Group therapy outcome measures

A repeated measures ANOVA of the positive SISST measured pre and post group therapy, revealed a significant effect *F*(1,21) = 41.53, *p* < 0.001, η = 0.66 with no group interaction *F*(1,21) = 0.20, *p* > 0.05, η = 0.009. A similar ANOVA of the negative SISST measured pre and post group therapy also revealed a significant effect *F*(1,21) = 42.63, *p* < 0.001, η = 0.67 with no group interaction *F*(1,21) = 0.10, *p* > 0.05, η = 0.005.

Repeated measures ANOVA yielded similar results for the FNE and LSAS. For the FNE, there was a significant effect of the group therapy *F*(1,21) = 22.90, *p* < 0.001, η = 0.52, with no group interaction effect *F*(1,21) = 1.62, *p* > 0.05, η = 0.07.

For the LSAS, there was also a significant effect of the group therapy *F*(1,21) = 19.72, *p* < 0.001, η = 0.48, with no group interaction *F*(1,21) = 1.09, *p* > 0.05, η = 0.004.

### Preconscious priming analyses

#### Effects of priming and control procedures on cognitions

**Figure 1:**
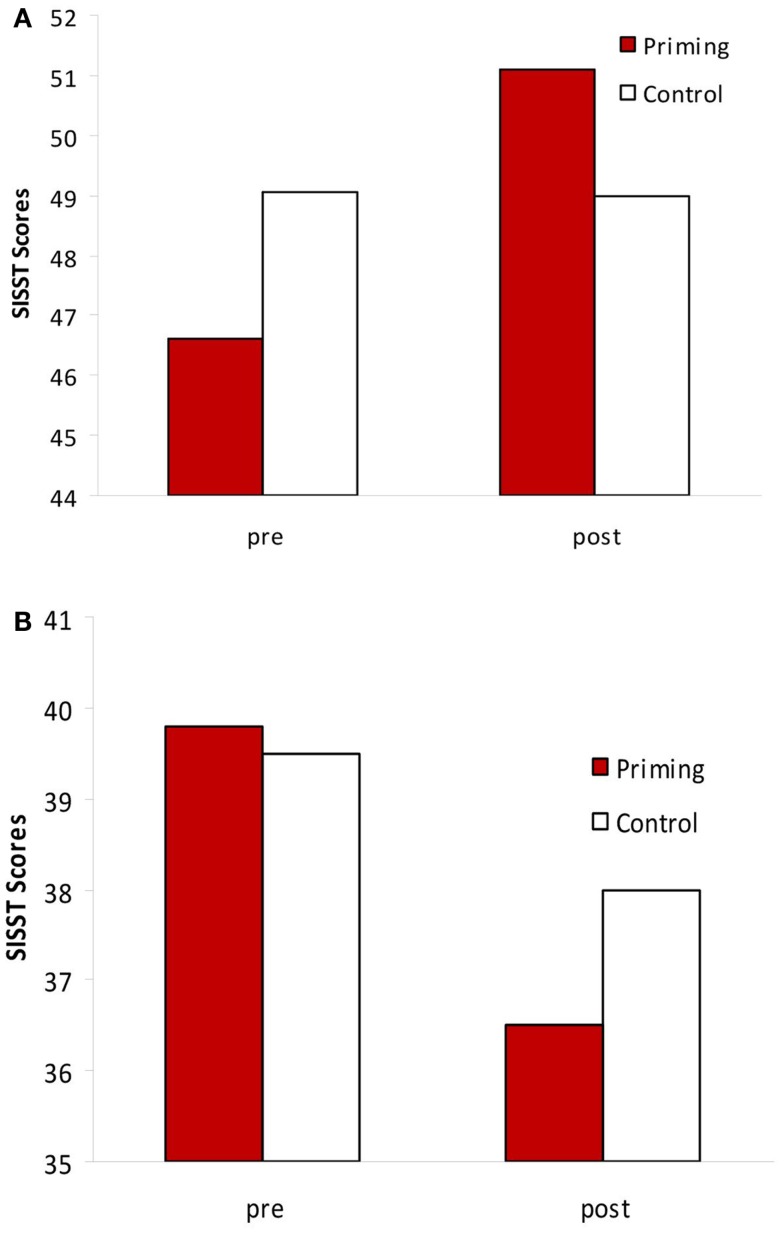
**(A)** SISST-positive statements changes before and after the priming vs. the control procedure. Priming: *t* = 3.1, *p* = 0.006; control: *t* = 0.86, ns. **(B)** SISST-negative statements changes before and after the priming vs. the control procedure. Priming: *t* = 2.18, *p* = 0.04; control: *t* = 1.1, ns.

To globally assess the effects of the preconscious priming procedure on social cognitions, paired *t*-tests were conducted to compared SISST scores before and after the 6-week priming for all subjects. The paired *t*-test on the positive SISST measures revealed a significant change pre and post priming *t*(18) = 3.1, *p* = 0.006 (Figure 1A). A change pre and post priming was also observed on the negative SISST scores *t*(17) = 2.18, *p* = 0.04 (Figure 1B). With the control procedure, there was no pre and post change neither on the positive items *t*(17) = 0.86, *p* > 0.05, or on the negative items *t*(17) = 1.10, *p* > 0.05.

#### ANOVAs

Since there was an unexplained nearly significant difference between the two groups (Priming first vs. Control first) at baseline for positive SISST scores, *t*(1,19) = 3.5, *p* = 0.07, an ANCOVA was performed to control for this baseline difference. The ANCOVA showed a main effect of time between weeks 12, 18, and 24, *F*(2,42) = 2.73, *p* < 0.10, η = 0.12, with no group interaction *F*(2,40) = 2.19, *p* > 0.05, η = 0.10.

**Figure 2:**
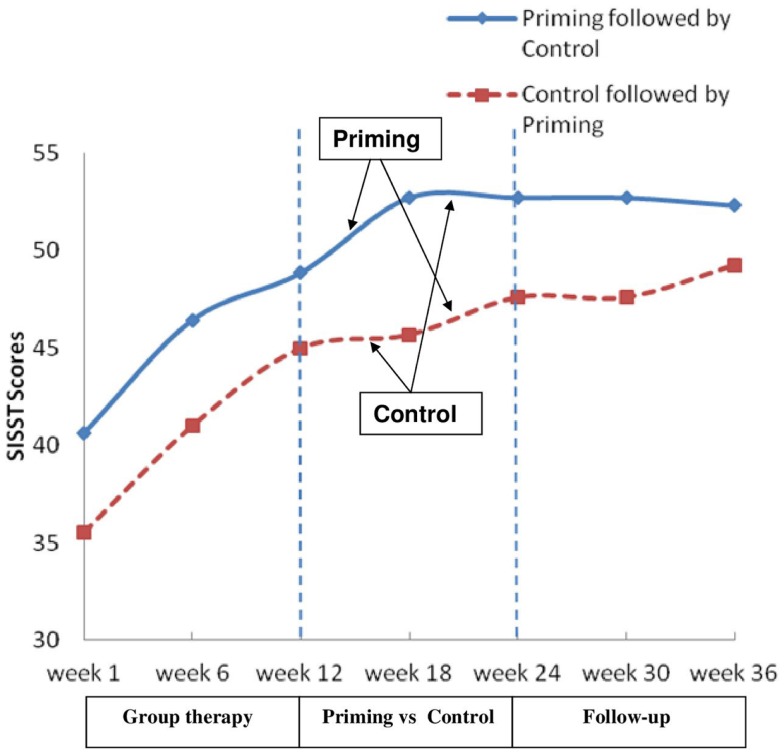
**Changes in SISST positive statements scores through the 36-weeks of the study**.

One-way ANOVA showed a significant interaction between the two groups at week 18 *F*(1,21) = 6.94, *p* < 0.05 and at week 24, *F*(1,21) = 5.38, *p* < 0.05 on the positive SISST scores (Figure 2).

There was no further difference between SISST scores at weeks 30 and 36, *F*(2,42) = 0.30, *p* > 0.05, η = 0.01 and no interaction effect *F*(2,42) = 0.78, *p* > 0.05, η = 0.04.

**Figure 3:**
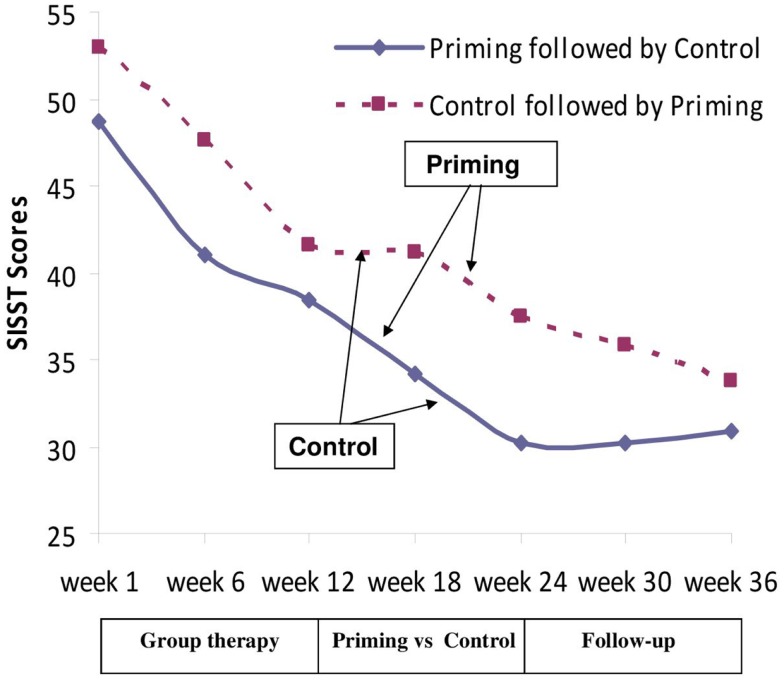
**Changes in SISST negative statements scores through the 36 weeks of the study**.

There was no baseline difference on negative SISST scores between the groups. A repeated measures ANOVA on the negative SISST items showed a main effect of time *F*(2,42) = 8.74, *p* < 0.01, η = 0.29 and no group interaction *F*(2,42) = 1.12, *p* > 0.10, η = 0.05 (Figure 3).

One-way ANOVA showed a significant interaction between the two groups between weeks 18 and 24, *F*(1,21) = 4.52, *p* < 0.04.

Repeated measures ANOVA on the FNE scores showed no significant time effect *F*(2,40) = 2.25, *p* > 0.10, η = 0.10 and no group interaction *F*(2,40) = 1.39, *p* > 0.10, η = 0.05. Repeated measures ANOVA on the LSAS scores showed a significant change between weeks 12 and 24, *F*(2,40) = 6.35, *p* < 0.004, η = 0.24 with no group interaction effect *F*(2,40) = 1.37, *p* > 0.10, η = 0.06.

### Ipsative analysis between time points

The significant interaction between the two sequences was confirmed by an ipsative analysis which compared the changes between each time measure. The analysis showed one significant difference between the two sequences between weeks 12 and 18 on positive SISST scores, that is when priming was compared to Control immediately after group therapy [*F*(1,17) = 10.53, *p* < 0.005].

### Difference related to high vs. low fear of negative evaluation

**Figure 4:**
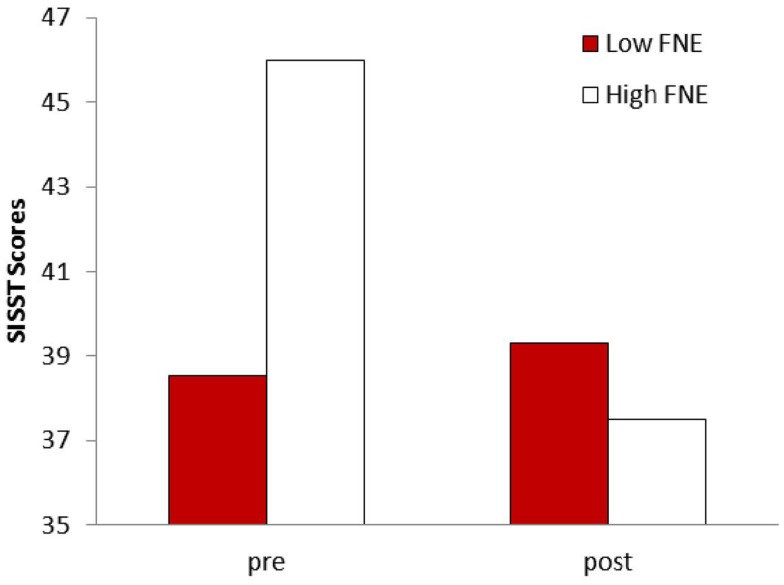
**Comparison of patients with lower FNE (fear of negative evaluation) scores at the end of group therapy vs. patients with higher FNE scores on their response to preconscious priming of SISST-negative statements: *F*(1,12) = 14.08, *p* = 0.003**.

To examine whether having a low or a high level of residual anxiety after group therapy modified the response to priming, we compared patients showing a high vs. a low FNE score at the end of group therapy. The whole group of patients was split by the median post therapy FNE scores and their SISST responses to preconscious priming were compared. Repeated measure ANOVA showed a significant difference between low and high FNE subgroups on negative SISST scores changes, *F*(1,12) = 14.08, *p* = 0.003, η = 0.54, but not for positive SISST items, *F*(1,12) = 0.80, *p* > 0.05, η = 0.06 (Figure 4).

## Discussion

The main conclusion is that an effect of preconscious priming was observed on social phobia related cognitions. Indeed, in contrast to the control procedure, preconscious priming resulted in increased positive cognitions or self statements and less negative cognitions related to social interactions as measured by the SISST, thus reflecting changes in the expected and coherent directions. Throughout this study, the proposed strategy has been referred to as preconscious priming because our team became convinced that priming and masked priming phenomena could provide an explanatory and parsimonious theory for what we were trying to achieve by repeated preconscious stimulations: rendering a set of positive cognitions, attitudes, and emotions more available to patients undergoing CBT or behavior therapy. However priming and masked priming, borrowed from experimental strategies used mainly to study word recognition, have never, to our knowledge, been applied to psychotherapy. But if priming can make words more readily available in laboratory word recognition tasks, it could logically produce similar effects daily in natural settings. Hence the results reported above suggest that a repeated activation or priming of cognitions, attitudes, and emotions can also render a useful semantic and affective network more readily available to a patient.

If priming and preconscious priming constitute a plausible and useful theory to describe and explain what occurred, alternative explanations could probably be suggested. For instance, research based on subliminal mere exposure effects (Bornstein, 1992) has demonstrated that simple exposure to a stimulus can enhance one’s attitude toward that stimulus in the direction of a more positive or more familiar judgment. Interestingly stimulus awareness has been shown to inhibit the mere exposure effect. But are the mechanisms behind the subliminal exposure effects very different from those explaining preconscious priming? It can be argued that they are probably similar and that they both indicate that repeated presentations of stimuli beyond the level of awareness tend to make them a more likely alternative to select.

Can the results reported be described as psychotherapy augmentation? The initial objective in developing that strategy was to augment the effects of psychotherapy for patients achieving limited progress in spite their efforts to change through an appropriate psychotherapy process. To evaluate if that was occurring, half the subjects with the best progress during the group therapy measured by FNE scores was compared to the other half with higher residual FNE scores. The patients with higher residual FNE ratings showed a significant diminution of their negative social anxiety related cognitions which was not the case for those with lower FNE. That finding supports the concept of psychotherapy augmentation: the preconscious priming seems to have rendered the cognitions less negative for the more fearful patients. Obviously, this split-half comparison provides only preliminary evidence due to the limited number of patients that could be compared. A study involving specifically patients with residuals symptoms post therapy could clarify this question.

The rapidity of change after the group therapy was different for the procedure of preconscious priming as compared with the control placebo procedure. Was it an augmentation or boosting effect that would not have taken place without the procedure or rather an acceleration of a change that would have occurred anyway following the psychotherapy? The cross-over design used in the study precludes a clear answer to that question since all patients received the preconscious priming intervention. However the results indicate that the sequence or the timing of the intervention influenced patients’ responses suggesting that an early priming procedure could be more effective. Indeed the improvement of cognitions related to social interactions was larger when preconscious priming was performed immediately after CBT than 6 weeks later. One of many unexplored issues concerns the possible effects of a simultaneous combination of CBT and preconscious priming.

One of the limits of this study is linked to the necessary rigid uniformity requested by a research design that probably played against finding positive effects of the procedure increasing the risk of an eventual type 2 error. Indeed a purely clinical context would have allowed much more flexibility: for instance, in the choice of the masking music that could have been adapted to patients’ preferences or changed periodically to increase their motivation. In the actual study, there was only one music for 12 weeks of twice a day listening and several patients reported being somewhat bored with that music when they shifted to the second CD, a fact that could have influenced their response. The experimental design requested also the choice of a single therapeutic focus throughout the procedure while a clinical context would allow an evolution or even a modification of that focus according to patients’ responses.

The procedure of preconscious priming applied in this study followed six steps and was highly personalized. Clearly each step carries a therapeutic potential. Defining a pertinent and feasible focus requires some amount of prior psychotherapeutic work. For that reason, the priming strategy is proposed as an addition to an ongoing or terminating therapy and does not constitute a therapy by itself. The steps concerned with focus selection, establishment of a semantic and affective network and writing formulations of the desired cognitions and attitudes can be seen as a form of condensed cognitive therapy. Using more general goals or formulations for large groups of patients would probably make the procedure meaningless from a cognitive therapy perspective where cognitive restructuring has to be tailored to each patient’s cognitive style, values, and even vocabulary. Thus the procedure described here can appear time-consuming which was true in the reported study. However our group is working on a computerized form of the procedure to facilitate and link the six steps, from the wording of the formulations that could be eased by the accumulating experience to their recording, their mixing with music and their delivery to patients in the form of a CD or through internet. Such a programed procedure should make the whole process more simple and user-friendly.

If the encouraging results reported here are confirmed in future research, it could suggest a most useful clinical application of preconscious priming. Such a priming procedure could be used for instance as a self administered addition to a therapy with the objective of extending the process beyond sessions or maintaining achieved cognitive change or even with the objective of shortening the therapy when the number of sessions has to be limited for practical reasons. Or, as argued here, it could be seen as an augmentation strategy when progress remains limited to help a patient switch from problematic cognitions and attitudes to more favorable ones.

## Conflict of Interest Statement

The authors declare that the research was conducted in the absence of any commercial or financial relationships that could be construed as a potential conflict of interest.
